# Regulation of primate lentiviral RNA dimerization by structural entrapment

**DOI:** 10.1186/1742-4690-5-65

**Published:** 2008-07-17

**Authors:** Tayyba T Baig, Christy L Strong, J Stephen Lodmell, Jean-Marc Lanchy

**Affiliations:** 1 Division of Biological Sciences, The University of Montana, Missoula, MT, 59812, USA

## Abstract

**Background:**

Genomic RNA dimerization is an important process in the formation of an infectious lentiviral particle. One of the signals involved is the stem-loop 1 (SL1) element located in the leader region of lentiviral genomic RNAs which also plays a role in encapsidation and reverse transcription. Recent studies revealed that HIV types 1 and 2 leader RNAs adopt different conformations that influence the presentation of RNA signals such as SL1. To determine whether common mechanisms of SL1 regulation exist among divergent lentiviral leader RNAs, here we compare the dimerization properties of SIVmac239, HIV-1, and HIV-2 leader RNA fragments using homologous constructs and experimental conditions. Prior studies from several groups have employed a variety of constructs and experimental conditions.

**Results:**

Although some idiosyncratic differences in the dimerization details were observed, we find unifying principles in the regulation strategies of the three viral RNAs through long- and short-range base pairing interactions. Presentation and efficacy of dimerization through SL1 depends strongly upon the formation or dissolution of the lower stem of SL1 called stem B. SL1 usage may also be down-regulated by long-range interactions involving sequences between SL1 and the first codons of the *gag *gene.

**Conclusion:**

Despite their sequence differences, all three lentiviral RNAs tested in this study showed a local regulation of dimerization through the stabilization of SL1.

## Background

The 5' untranslated region of the lentiviral genomic RNA is replete with RNA signals involved in different stages of the replication cycle, such as transcription transactivation, polyadenylation, tRNA primer binding, dimerization, encapsidation, splicing, and translation [1]. The RNA signals mediate viral functions through RNA-protein (genomic RNA encapsidation and reverse transcription) and RNA-RNA interactions (dimerization, tRNA hybridization to PBS). Although most of these signals can be linked to a precise stage of the viral replication cycle, they overlap structurally and functionally ([2-4]). For instance, the stem-loop 1 (SL1) dimerization signal overlaps with the genomic RNA encapsidation signal in HIV-2 ([5-8]). Another interesting characteristic of retroviral leader RNA signals is the fact that their presentation may vary during the different stages of viral replication. For example, the dimerization and encapsidation signals in Moloney murine leukemia virus genomic RNA are proposed to be initially masked and need to be activated by Gag protein-dependent RNA structural rearrangements [9].

Electron microscopy studies of packaged genomic RNAs revealed that the two RNA molecules are strongly associated with each other through their 5' ends, termed the dimer linkage structure ([10,11]). In HIV-1, a short sequence located in the 5' untranslated region that promotes dimerization of partial leader transcripts was identified and named the dimerization initiation site (DIS) or stem-loop 1 (SL1) ([12-14]). The SL1 element maintains two RNA molecules in a dimeric state *in vitro*, either through a kissing-loop interaction, or an extended duplex base pairing arrangement (for review, see [2-4]). Roles of SL1 in the different stages of viral replication have been characterized in cell culture (reviewed in [2], [15]). Initially proposed from *in vitro *results [12], the genomic RNA dimerization initiation role of SL1 was recently confirmed in HIV-1 [16]. A riboswitch model was proposed in which extensive structural rearrangements of the whole leader region could influence the presentation of SL1 (and other RNA signals) and thus dimerization and packaging efficiencies [3]. In this model the HIV-1 leader RNA can adopt two different conformations: a long-distance interaction (LDI) between the polyA signal and SL1 domains and a distinct branched multiple-hairpin structure (BMH) [17]. Several predicted conformations of the leader region include base pairing between a C-rich sequence in the 5' part of U5 and a G-rich sequence around the *gag *translation initiation region [17]. Here we call this interaction CGI, for C-box – G-box interaction, to specifically indicate this base pairing rather than an overall conformation of the leader RNA such as LDI or BMH. Formation of CGI favors the BMH conformer in HIV-1 and thus promotes the presentation of SL1 as an efficient dimerization signal ([17,18]).

**Figure 1:**
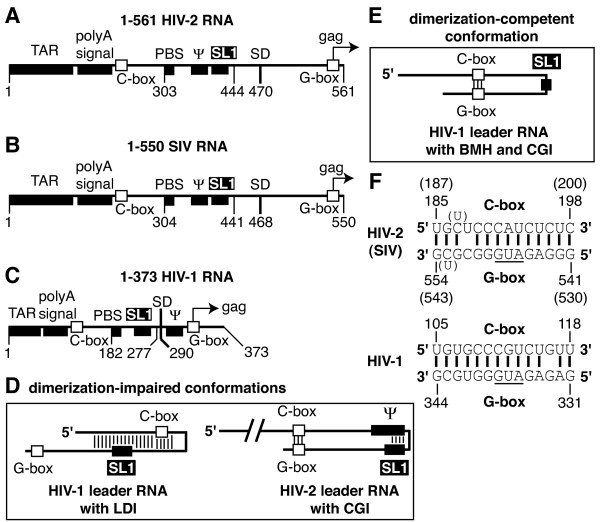
**5' leader region of lentiviral genomic RNA and proposed structural conformations**. The landmark sequences with known functions of HIV-2 (**A**), SIV (**B**), and HIV-1 (**C**) leader RNAs are indicated by boxes with the names indicated above. TAR, polyA signal, PBS, Ψ, SL1, SD, and *gag *represent the trans-activation region, the polyA signal domain, the primer-binding site, the major encapsidation signal, the stem-loop 1, the major splice donor site, and the 5' end of the Gag protein coding region, respectively. The numbering corresponds to the genomic RNA position. HIV-2 and SIV Ψ sequences correspond to nts 380–408, and 381–409, respectively ([8,53]). HIV-1 Ψ is represented by the stem-loop 3 structure (nts 312–325, [54]). **D**. Schematics of the proposed conformations of leader RNAs that impair SL1-mediated dimerization are shown. HIV-1 leader RNA can form an extensive, long-range base pairing interaction between the polyA signal and SL1 domains (called LDI) [17]. HIV-2 leader RNA can make a long-range base pairing interaction (called CGI for C-box – G-box interaction) between a sequence located between the polyA and PBS domains and another one located at the *gag *translation initiation region (C-box and G-box, respectively). Formation of CGI impairs SL1-mediated dimerization in HIV-2 ([21,25]). **E**. The proposed conformation of HIV-1 leader RNA that favors dimerization is characterized by a distinct branched multiple-hairpin structure (BMH) that contains an intact CGI [17]. **F**. Representation of HIV-2/SIV (top) and HIV-1 (bottom) CGI base pairing models ([17,25]). Numbers and nucleotides in parentheses represent the SIV equivalent of HIV-2 numbers and nucleotides. The translation initiation codon of the *gag *gene is underlined.

*In vitro*, two dimerization signals exist within the HIV-2 genomic RNA leader region ([19-22]). One signal involves the 5' end of the tRNA-primer binding site (PBS) ([19,20]) and exhibits properties of loose dimerization (previously defined in [23,24]). Because it overlaps with the PBS, its role in viral replication, if any, could only occur before the hybridization of the tRNA primer during the formation of the viral particle ([19,20]). Although the second HIV-2 dimerization signal is homologous to the element SL1 in HIV-1 leader RNA, its efficiency as a tight dimerization element is suboptimal in large HIV-2 RNA constructs encompassing the whole 5' untranslated leader region ([19-21]). We and others showed that the impaired tight dimerization phenotype correlated with the formation of the CGI long-range base pairing interaction (Figure 1) ([21,25]). We proposed that, contrary to HIV-1, formation of CGI favors intramolecular entrapment of SL1 and thus decreases its use as a tight dimerization signal in HIV-2 RNA ([25,26]). Indeed, an *in vitro *evolution analysis revealed that the SL1 region adopts discrete conformations with different dimerization abilities [27]. Compared to HIV-1 and HIV-2, little is known about RNA dimerization in SIVs [19].

To determine whether common mechanisms of SL1 regulation exist among divergent lentiviral leader RNAs, we compared the dimerization properties of SIVmac239, HIV-1, and HIV-2 leader RNA fragments using homologous constructs and experimental conditions. Overall, it appears that SIV, HIV-1, HIV-2 RNAs have several common features with regard to SL1-mediated dimerization, which points to conserved regulation mechanisms by homologous RNA structures. Truncation analysis revealed that tight dimerization of the three lentiviral leader RNAs is modulated by interactions of nucleotides located between SL1 and the first codons of the *gag *open reading frame that affect both SL1 presentation and overall leader conformation. Most important, all three lentiviral RNAs tested in this study demonstrated a local regulation of dimerization through the stabilization of SL1 by its lower stem structure called stem B.

## Methods

### Template construction for *in vitro *transcription

A sense primer containing a *Bam*HI site and the promoter for the T7 RNA polymerase and an antisense primer containing an *Eco*RI site (Table 1) were used to amplify the first 437, 444, or 561 nucleotides of HIV-2 genomic RNA sequence, ROD isolate (nt 1 of the genomic RNA sequence corresponds to nt 1 of [GenBank:M15390]), and the first 435, 441, or 550 nucleotides of SIV genomic RNA sequence, mac239 isolate (nt 1 of the genomic RNA sequence corresponds to nt 775 of [GenBank:M33262]). The extended stem B mutation was introduced into a 1–444 HIV-2 ROD sequence using a modified antisense primer (HIV2 asMUT444Eco, Table 1). This mutation is a substitution of nts G^437^-T^438 ^by the sequence CTTTCTA. DNA template plasmids containing the first 272, 277, or 373 nucleotides of HIV-1 NL4-3 genomic RNA and a T7 RNA polymerase promoter were constructed using a similar strategy (nt 1 of the genomic RNA sequence corresponds to nt 455 of [GenBank:AF324493]). The numbers used to define RNA constructs (for instance 1–561 HIV-2 RNA) are based on genomic RNA numbering. The HIV-2 ROD DNA template (modified plasmid pROD10) was provided by the EU Programme EVA/MRC Centralised Facility for AIDS Reagents, NIBSC, UK (Grant Number QLK2-CT-1999-00609 and GP828102). The SIV mac239 (p239SpSp5' plasmid) and HIV-1 (p83-2 plasmid) DNA templates were obtained from Dr. Ronald Desrosiers through the AIDS Research and Reference Reagent Program ([28-30]). The digested polymerase chain reaction products were cloned in the *Bam*HI and *Eco*RI sites of the pUC18 plasmid.

**Table 1:** Cloning oligonucleotides used in this study. The 5' to 3' sequence is indicated from left to right.

HIV-2 sT7Bam	TAG GAT CCT AAT ACG ACT CAC TAT AGG TCG CTC TGC GGA GAG
HIV-2 as437Eco	AAG AAT TCA CGC TGC CTT TGG TAC CTC GGC C
HIV-2 as444Eco	AAG AAT TCG CTC CAC ACG CTG CCT TTG
HIV-2 asMUT444Eco ^a^	TTG AAT TCG CTC CTA GAA AGA CGC TGC CTT TGG TAC CTC G
HIV-2 as561Eco	AAG AAT TCA GTT TCT CGC GCC CAT CTC CC
SIV as435Eco	AAG AAT TCA CGC CGT CTG GTA CCG
SIV as441Eco	TTG AAT TCG CTC CTC ACG CCG TCT GG
SIV as550Eco	AAG AAT TCA GTT TCT CAC GCC CAT CTC CC
HIV-1 sT7Bam	TAG GAT CCT AAT ACG ACT CAC TAT AGG TCT CTC TGG TTA GAC C
HIV-1 as272Eco	TTG AAT TCT CTT GCC GTG CGC GCT TCA GC
HIV-1 as277Eco	TTG AAT TCT CGC CTC TTG CCG TGC G
HIV-1 as373Eco	TTG AAT TCT CCC CCG CTT AAT ACC GAC

a: this oligonucleotide was used to construct the 1–444 HIV-2 RNA with an extended SL1 (Figure 6C).

### RNA synthesis and purification

The different plasmids were linearized with *Eco*RI prior to *in vitro *transcription. RNAs were synthesized by *in vitro *transcription of the *Eco*RI-digested plasmids with the AmpliScribe T7 transcription kit (Epicentre). After transcription, the DNA was digested with the supplied RNase-free DNase, and the RNA was purified by ammonium acetate precipitation followed by size exclusion chromatography (Bio-Gel P-4, Bio-Rad).

### *In vitro *dimerization of SIV, HIV-1, and HIV-2 RNAs

**Figure 2:**
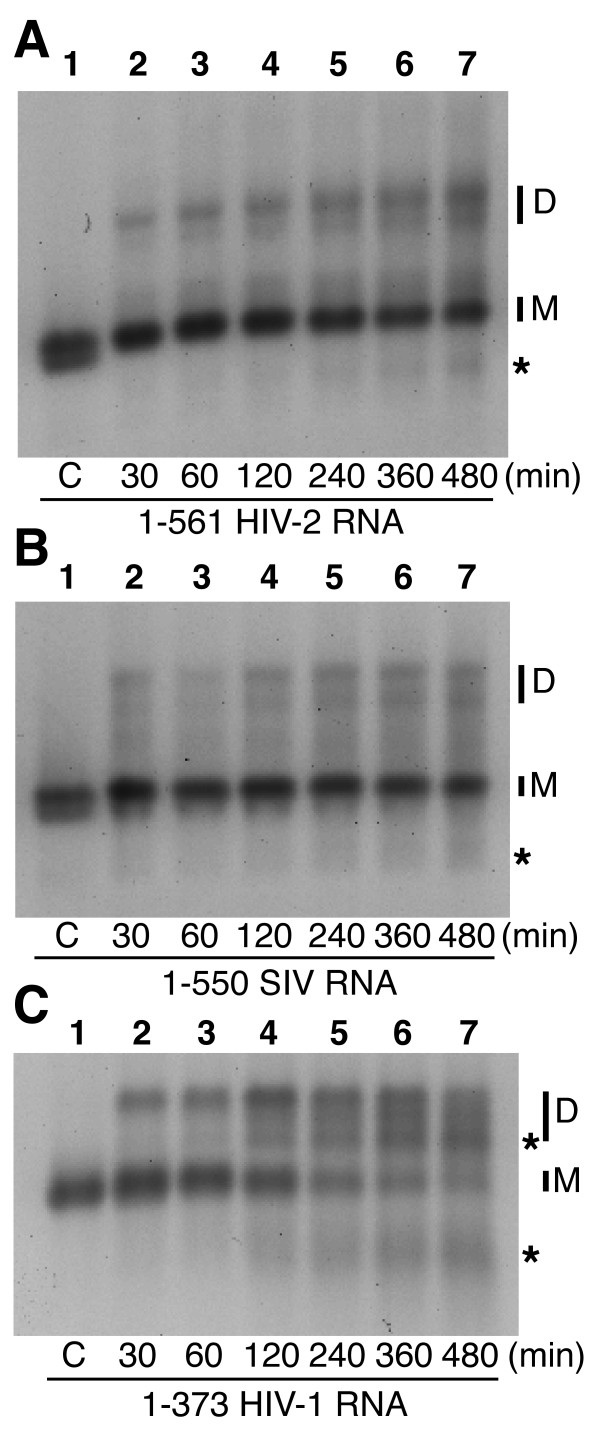
**Kinetics of tight dimerization of HIV-2, SIV and HIV-1 leader RNA fragments**. 1–561 HIV-2 ROD isolate RNA (**A**), 1–550 SIV mac239 isolate RNA (**B**), and 1–373 HIV-1 NL4-3 isolate RNA (**C**) were incubated for 0.5–8 hours at 55°C in dimer buffer. After incubation, samples were subjected to electrophoresis on TBE agarose at 26°C during which only tight dimers and magnesium-independent conformers remain intact. In order to load all incubations at the same time, the incubations were initiated in reverse order, *i.e*. the longest incubation first. The monomer and dimer RNA species are indicated by M and D, respectively. Fast-migrating bands are indicated by asterisks. Lane 1 in each panel represents monomeric RNA that was denatured at 90°C, then quenched on ice immediately prior to loading (C for control).

Five to eight pmol of RNA were denatured in 8 μl water for 2 minutes at 90°C and quench cooled on ice for 2 minutes. After the addition of 2 μl buffer M (HIV-1 dimer buffer; final concentrations: 50 mM Tris-HCl pH 7.5 at 37°C, 100 mM KCl, 1 mM MgCl_2_) or buffer H (HIV-2 or SIV dimer buffer; final concentrations: 50 mM Tris-HCl pH 7.5 at 37°C, 300 mM KCl, 5 mM MgCl_2_), dimerization was allowed to proceed for 30 minutes or up to eight hours at 55°C. The optimal tight dimerization conditions for HIV-2 require high salt buffer [25]. The optimal HIV-1 tight dimerization conditions are broader and tight dimers can form in lower salt conditions ([24,31]). Here we have used medium salt buffer for the HIV-1 dimerization to make the dimerization conditions homologous to the HIV-2/SIV conditions. We used 55°C since the incubation of HIV-1 and HIV-2 RNAs at 55°C allows formation of SL1-mediated tight dimers ([20,23,24]). In order to load all incubations at the same time, long extended incubations (Figure 2) were started in inverted order, that is, the longest incubation first. To avoid volume changes of the dimerization mixture due to water condensation under the lid of the reaction tube, the long extended incubations were done at 55°C in a PCR machine with heating lid. When assaying the early stages of dimerization with large RNA constructs, we used 30 min as standard incubation time [26].

The samples were then cooled on ice to stabilize dimers formed during incubation and loaded on a 0.8% agarose gel with 2 μl glycerol loading dye 6× (40% glycerol, Tris-borate 44 mM pH 8.3, 0.25% Bromophenol blue). Electrophoresis was carried out at 3 V/cm for 2 hours at room temperature (26°C) in Tris-borate 44 mM pH 8.3, EDTA 1 mM (TBE). After electrophoresis, the ethidium bromide-stained gel was scanned on a Fluorescent Image Analyzer FLA-3000 (Fujifilm).

### Analysis of regulatory RNA signals using antisense oligonucleotides

Five pmol of RNA with or without 100 pmol of oligonucleotide (Table 2) were denatured in 8 μl water for 2 minutes at 90°C and quench cooled on ice for 2 minutes. After the addition of 2 μl fivefold concentrated dimer buffer, dimerization was allowed to proceed for 30 minutes at 55°C. The samples were then cooled on ice to stabilize dimers formed during incubation and loaded on a 0.8% TBE agarose gel. Electrophoresis was carried out for 90 minutes at 26°C and 3 V/cm. After electrophoresis, the ethidium bromide-stained gel was scanned on a Fluorescent Image Analyzer FLA-3000 (Fujifilm).

**Table 2:** Antisense oligonucleotides used in this study. The 5' to 3' sequence is indicated from left to right.

HIV-2 asΨ	CTA GGA GCA CTC CGT CGT GGT TTG
HIV-2 asSL1	TGG TAC CTC GGC CCG CGC CT
HIV-2 asC	CTA GGA GAG ATG GGA GTA CAC AC
HIV-2 asG	CAT CTC CCA CAA TCT TCT ACC
SIV asΨ	ATA GGA GCA CTC CGT CGT GGT TGG
SIV asSL1	TGG TAC CGA CCC GCG CCT
SIV asC	CTA GGA GAG ATG GGA ACA CAC AC
SIV asG	CAT CTC CCA CTC TAT CTT ATT ACC CC
HIV-1 as246	CGA GTC CTG CGT CGA GAG ATC TCC
HIV-1 asSL1	TGC GCG CTT CAG CAA GCC GAG TCC
HIV-1 asC	AGA CGG GCA CAC ACT ACT TTG AGC A
HIV-1 asG	CAT CTC TCT CCT TCT AGC CTC

### Kinetics of tight dimer formation

Fifty pmol of RNA with or without one nmol of antisense oligonucleotide (Table 2) were denatured in 80 μl water for 2 minutes at 90°C and quench cooled on ice for 2 minutes. After the addition of 2 μl 5× dimer buffer under the lid of 10 tubes, 8 μl of denaturated RNA was aliquoted to each tube. The dimerization was started by a 5 second spin in a bench top centrifuge followed by immediate loading of the tubes in a heating block at 55°C. Dimerization was allowed to proceed for 2 to 16 minutes. At each time point a tube containing 10 μl of reaction mixture was removed from the heating block, mixed with 2 μl glycerol loading dye 6× and loaded on a 0.8% agarose TBE gel. Electrophoresis was carried out at room temperature (26°C) and 3 V/cm. After electrophoresis, the ethidium bromide-stained gel was scanned on a Fluorescent Image Analyzer FLA-3000 (Fujifilm). Quantification of the extent of dimerization was performed using Fujifilm Image Gauge V4.22 software. The data were fitted using a second order conformation model [32]:

1Mt=1M0+2kdim⁡t

where M_t _is the concentration of monomer at time t, M_0 _is the initial concentration of dimerization-competent monomer, and k_dim _is the second order rate constant of dimerization (μM^-1^min^-1^).

### Effect of the dimerization incubation temperature

Five pmol of RNA were denatured in 8 μl water for 2 minutes at 90°C and cooled on ice for 2 minutes. After addition of 2 μl 5× dimer buffer, dimerization was allowed to proceed for 30 minutes at 24°, 37°, 40°, 45°, 50°, 55°, or 60°C. The samples were then loaded on a 0.8% agarose TBE gel. Electrophoresis was carried out for 2 h at 26°C and 3 V/cm. After electrophoresis, the ethidium bromide-stained gel was scanned on a Fluorescent Image Analyzer FLA-3000 (Fujifilm).

### Prediction of secondary structures

Mfold version 3.2 was used to predict the most stable secondary structures for the SL1 region of HIV-2 ROD, SIV mac239, and HIV-1 NL4-3 RNAs. The software used is found on the mfold server . The ΔGs at 37°C of the most stable conformation for lentiviral SL1s with or without stem B were recorded in Table 3.

**Table 3:** Calculated ΔG values (kcal/mol at 37°C) for lentiviral SL1 region folding.

	SL1 version	nts folded	ΔG
HIV-2	short	408–436	-10.2
HIV-2	stem B	392–444	-18.2
HIV-2	extended stem B	392–449 ^a^	-36.4
SIV	short	409–433	-7
SIV	stem B	393–445	-15.9
HIV-1	short	248–270	-6.7
HIV-1	stem B	243–277	-10.9 ^b^

The indicated energies are given for the most stable (initial ΔG value) secondary structure.

a: nts 437 and 438 have been substituted by seven nts (Figure 6C). Thus nt 444 would be numbered 449 in this mutated sequence.

b: The indicated energy is given for the structure described in Figure 5C that was obtained with a constrained 243–277 base pair. Without constraint, the lower part of stem C in the most stable structure folds with a 243–272 base pair (as studied in [48]).

## Results

### Tight dimerization characteristics of HIV-2, SIV, and HIV-1 leader RNAs

The ability of 1–561 HIV-2, 1–550 SIV, and 1–373 HIV-1 RNAs to form tight dimers when incubated at 55°C in dimerization buffers was compared. All three RNA constructs encompass the 5' untranslated leader region and the first several codons of the *gag *open reading frame of the genomic RNA (Methods, Figure 1). Tight dimers withstand dissociation during semi-native gel electrophoresis (Tris-borate EDTA (TBE) buffer at 26°C). By contrast, loose dimers are detected only by using native conditions, since they dissociate upon loading in semi-native TBE/26°C gels [33]. Loose dimers are thought to correspond to a loop-loop interaction between two SL1 motifs, whereas tight dimers are thought to represent a more extensive SL1–SL1 interaction due to the intra- to intermolecular conversion of the stems located below the SL1 loop ([2,4]).

The tight dimerization of HIV-2 1–561 RNA was similar to what has been previously described ([19,20]). Only a minor fraction of RNA molecules dimerized after 30 or 60 min (Figure 2A, compare lane 1 to lanes 2 and 3). Indeed, the dimerization yield was less than 50% after an eight hour incubation at 55°C (Figure 2A, lane 7). The tight dimerization of 1–550 SIV RNA was similar to HIV-2, with a slightly lower level of tight dimers formed after extended incubation (Figure 2B).

The formation of HIV-1 RNA dimers, measured by the disappearance of the monomer RNA species, was twice as efficient as HIV-2 or SIV over the whole eight hour experiment, although the interpretation of longer time points was made more difficult by the appearance of two other RNA species that appeared after two hours. One band migrated between the monomer and dimer bands and another one was below the monomer band (Figure 2C). Though we cannot rule out that a small fraction of these RNA species represent fast-migrating conformers, electrophoretic analysis of these reactions under denaturing conditions revealed that the majority of these RNA species represent cleaved RNA molecules (data not shown). Significant cleavage was not observed in the HIV-2 or SIV RNAs during extended incubation (Figure 2), which allowed us to rule out an obvious RNase contamination, since most of the buffers, transcription components were shared between all RNAs tested in this study.

The relative inefficiency of HIV-2 and SIV RNA tight dimerization suggested that the presentation of SL1 was not optimal in these RNAs. Though more efficient than HIV-2 and SIV, the yield of HIV-1 tight dimerization during the first hour was low (Figure 2C). Since SL1 presentation is modulated by short- and long-range interactions in HIV-2 ([21,25-27]) or HIV-1 [34], we hypothesize that co-incubation of lentiviral leader RNAs with antisense oligonucleotides directed against these modulating interactions may improve SL1 presentation and thus increase tight dimerization.

### Analysis of long-range regulatory interactions using antisense oligonucleotides

**Figure 3:**
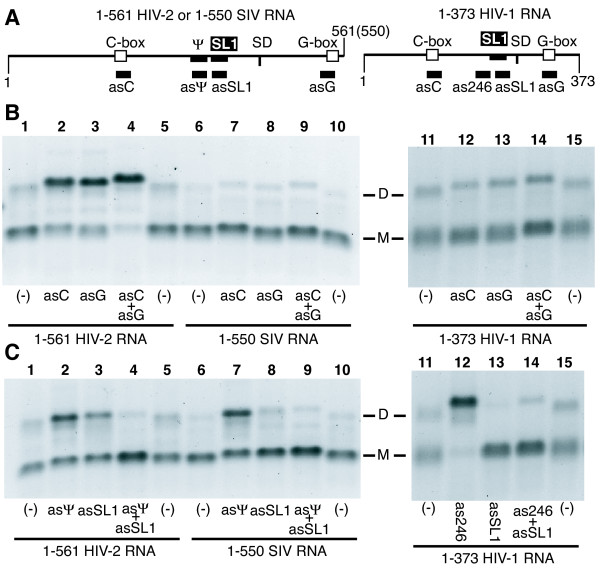
**Antisense oligonucleotide-mediated activation or suppression of HIV-2, SIV and HIV-1 RNA tight dimerization**. **A**. Schematic representation of the leader RNAs as described in Figure 1 with the oligonucleotide binding sites indicated (left panel, HIV-2 or SIV, right panel, HIV-1). **B**. 1–561 HIV-2 (left), 1–550 SIV (center) and 1–373 HIV-1 (right) RNAs were incubated at 55°C for 30 minutes in their respective dimer buffers with or without a 20-fold molar excess of antisense oligonucleotides directed against the C-box or G-box elements (Table 2) before loading on a TBE/26°C agarose gel. The 5' end of all asG oligonucleotides bind to the start codon of the *gag *gene in the G-box element and thus to the majority of nucleotides involved in the CGI structures (8 nts out of 13, Figure 1F). The asC oligonucleotides bind to all (HIV-2 and SIV) or most (HIV-1, 10 nts out of 13) of lentiviral C-box elements (Figure 1F). Lanes 1, 5, 6, 10, 11 and 15 correspond to RNA incubated in dimer buffer at 55°C without oligonucleotide. The monomer and dimer RNA species are indicated by M and D, respectively. **C**. 1–561 HIV-2 (left), 1–550 SIV (center) and 1–373 HIV-1 (right) RNAs were incubated at 55°C for 30 minutes in dimer buffer with or without a 20-fold excess of antisense oligonucleotides directed against sequences upstream of SL1 and including stem B (asΨ in HIV-2 and SIV, as246 in HIV-1) or the loop and 5' stem of SL1 (asSL1) before loading on a TBE/26°C agarose gel.

SL1-mediated dimerization can be regulated by long-range interactions (LDI in HIV-1 [35] or C-box – G-box interaction (CGI) in HIV-1 and HIV-2 ([21,25,35]) and short-range interactions (Ψ encapsidation signal in HIV-2 [26]) (Figures 1D, 1E and 1F). To determine the relative importance of short- and long-range interactions in modulating SL1-mediated tight dimerization of lentiviral RNAs, we tested the effects of antisense oligonucleotides directed against the known RNA elements that regulate SL1-mediated tight dimerization in HIV-2 [26]. We and others demonstrated that the impaired phenotype of HIV-2 tight dimerization correlated with the formation of the long-range CGI ([21,25]). The CGI is formed through base pairing of a sequence located in the 5' part of U5 and the *gag *translation initiation region (C-box and G-box, respectively, Figure 1F) ([21,25]). Antisense oligonucleotides directed against the C-box or G-box elements (asC and asG, respectively) greatly increased the dimerization yield of 1–561 HIV-2 RNA, but not 1–550 SIV RNA after a 30 min-incubation (Figure 3A, compare lanes 2–4 to 7–9).

Similar to SIV, the incubation of 1–373 HIV-1 RNA with the homologous oligonucleotides showed no effect (40% of tight dimers with or without oligonucleotides; Figure 3A, lanes 11 to 15). Experiments using radioactive oligonucleotides or RNase H cleavage assay [36] confirmed that the oligonucleotides bind the HIV-1 RNA effectively and at the expected locations (data not shown). The lack of effect of asC oligonucleotide binding on HIV-1 dimerization suggests that the formation of the LDI structure was minimal in our experiments. Likewise, the lack of effect of asG oligonucleotide binding suggests that disruption of the BMH structure did not promote either LDI or tight dimer formation. Thus, contrary to HIV-2, disruption of CGI with antisense oligonucleotides did not have an effect on SIV and HIV-1 RNA tight dimerization. This suggests that, besides CGI, stronger local regulatory interactions may impair the use of SL1 as a tight dimer element in SIV and HIV-1.

### Analysis of short-range regulatory interactions using antisense oligonucleotides

To test whether local base pairing in the SL1 region affects tight dimerization, we used an antisense oligonucleotide directed against the 5' stem B of SL1 and sequences upstream (as Ψ for HIV-2 and SIV, as246 for HIV-1, Table 2 and Figure 3A). Stem B represents the lower stem of the SL1 structure [14]. The impetus for this experiment came from previous observations of local regulation of SL1-mediated dimerization in HIV-2 RNA [26]. Incubation of HIV-2 1–561 RNA with an oligonucleotide binding to the loop and 5' stem of SL1 induced dimerization mediated by the 10-nt autocomplementary sequence (pal: 5'-392-GGAGUGCUCC-401) located in the HIV-2 encapsidation signal Ψ (Figure 3C, lane 3). Likewise, incubation of SIV and HIV-1 RNAs with the homologous asΨ and as246 oligonucleotides improved tight dimerization (Figure 3C, lanes 7 and 12). Co-incubation with the anti-SL1 oligonucleotide (asSL1, Table 2) confirmed that the increased tight dimerization was SL1-dependent (Figure 3C, lanes 4, 9, 14). Thus, the region upstream of SL1 including stem B has a negative effect on lentiviral tight dimerization. However, the different levels of tight dimers formed in the absence of oligonucleotides, (Figures 2 and 3) indicate that different SL1 sequences may also influence dimerization properties of lentiviral RNAs.

### Chimeric RNAs reveal SL1 loop sequence affects tight dimerization

**Figure 4:**
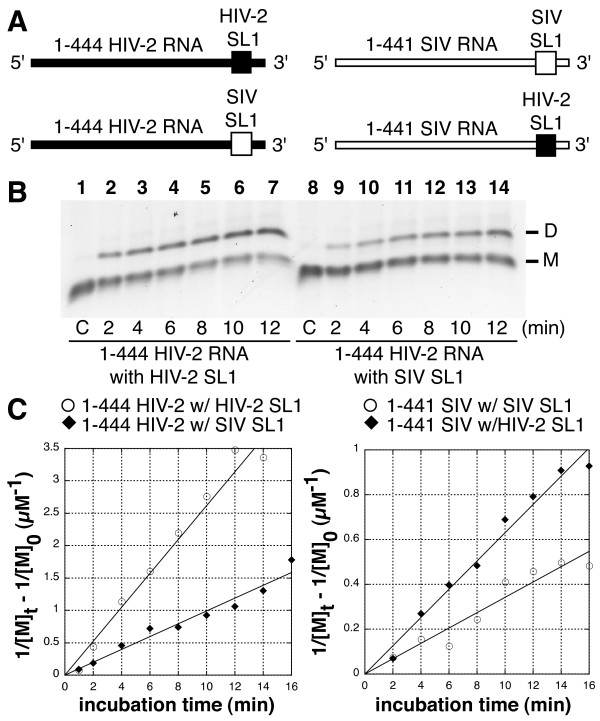
**Effect of SL1 replacement in HIV-2 and SIV RNA chimeras**. **A**. Schematic representation of HIV-2 and SIV SL1 with the substituted regions. HIV-2 and SIV sequences are represented with closed and open symbols, respectively. The SL1 sequences (nts 409 to 436) in 1–444 HIV-2 and 1–441 SIV (nts 410 to 433) RNAs were substituted by SIV and HIV-2 SL1 sequences, respectively. The SL1 sequences substituted encompass the apical loop and the stems C1 and C2. **B**. All four RNAs were individually incubated at 55°C in buffer H for 2, 4, 6, 8, 10, and 12 minutes before loading on a TBE/26°C agarose gel. A typical kinetics experiment with 1–444 HIV-2 RNA bearing wild type HIV-2 (lanes 1–7) or substituted SIV SL1 (lanes 8 to 14) is shown. Lane 1 represents monomeric RNA that was denatured at 90°C, then quenched on ice immediately prior to loading. **C**. Plots of the kinetic data for 1–444 HIV-2 (left panel) and 1–441 SIV (right panel) RNAs with wild type (open circles) or substituted SL1 (closed diamonds). The dimerization rate was extrapolated from the kinetics experiments according to [32]. The dimerization rate equals the slope of each linear curve divided by two (see Methods). For 1–444 HIV-2 RNA bearing wild type HIV-2 or substituted SIV SL1, k_dim _equals 0.11 ± 0.024 and 0.05 ± 0.007 μM^-1^min^-1^, respectively (duplicate experiments). For 1–441 SIV RNA bearing wild type SIV or substituted HIV-2 SL1, k_dim _equals 0.013 ± 0.004 and 0.026 ± 0.007 μM^-1^min^-1^, respectively (duplicate experiments).

While there is strong sequence homology between HIV-2 ROD and SIV mac239 isolates [37], their SL1 sequences differ, especially stem C and the loop, which could influence dimerization rates. To test if the SIV SL1 sequence might contribute to the impaired tight dimerization independently of CGI, we built chimeras between HIV-2 and SIV SL1 in the context of partial leader RNA fragments (1–444 and 1–441 RNAs for HIV-2 and SIV, respectively). We used smaller RNAs so that sequences between SL1 and the first codons of the *gag *gene would not influence tight dimerization directly or indirectly ([20,22]). To build chimeric RNAs, SL1 sequences in 1–444 HIV-2 (nts 409 to 436) and 1–441 SIV (nts 410 to 433) RNAs were substituted by the SIV and HIV-2 SL1 homologs, respectively. We measured the apparent tight dimerization rate at 55°C of all four RNA constructs (Figure 4). The wild type 1–444 HIV-2 RNA was the fastest to dimerize (k_dim _= 0.11 ± 0.024 μM^-1^min^-1^), while the wild type 1–441 SIV RNA was the slowest (k_dim _= 0.013 ± 0.004 μM^-1^min^-1^, Figure 4C). In fact, introducing SIV SL1 in HIV-2 RNA decreased the dimerization rate two-fold, and introducing HIV-2 SL1 in SIV RNA increased the dimerization rate two-fold (Figure 4).

Thus, the difference of only a few nucleotides in the SL1 sequence influences tight dimerization rates in SIV and HIV-2 RNAs. However, the incomplete correlation between the SL1 sequence and the dimerization yield in the chimeric RNAs indicates that other features of the leader RNA, like the stem B element, may influence the formation of tight dimers.

### Stem B affects the tight dimerization rate in lentiviral RNAs

**Figure 5:**
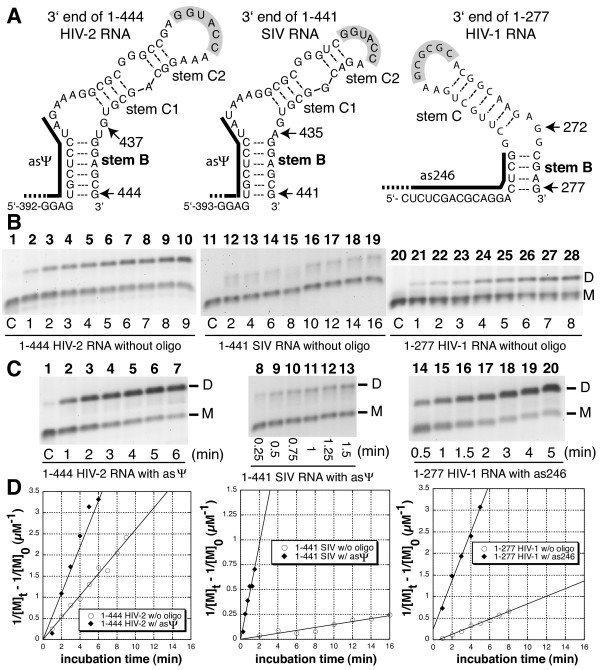
**Anti-stem B oligonucleotide-mediated activation of HIV-2, SIV and HIV-1 RNA tight dimerization**. **A**. Schematic representation of HIV-2, SIV, and HIV-1 SL1 domains. The black bars represent the binding sites of antisense oligonucleotides used in this experiment (Table 2). **B and C**. The time-dependent dimerization of 1–444 HIV-2, 1–441 SIV and 1–277 HIV-1 RNAs were measured at 55°C as described in Figure 4, without (B) or with (C) a 20-fold excess of antisense oligonucleotides directed against the sequence upstream of SL1 including the 5' side of stem B (A and Table 2). Typical kinetics experiments with 1–444 HIV-2 RNA (B and C, left panels), 1–441 SIV SL1 (B and C, middle panels), and 1–277 HIV-1 RNA (B and C, right panels) are shown. Lanes 1 (B and C), 11 and 20 (B) represent monomeric RNA that was denatured at 90°C, then quenched on ice immediately prior to loading. **D.** Plots of the kinetic data for RNAs incubated without (open circle) or with (closed diamonds) antisense oligonucleotides. The dimerization rate was extrapolated as described in Figure 4. For 1–444 HIV-2 RNA incubating without or with oligonucleotide, k_dim _equals 0.11 ± 0.024 and 0.3 ± 0.039 μM^-1^min^-1^, respectively (duplicate experiments). For 1–441 SIV RNA incubating without or with oligonucleotide, k_dim _equals 0.013 ± 0.007 and 0.24 ± 0.007 μM^-1^min^-1^, respectively (duplicate experiments). For 1–277 HIV-1 RNA incubating without or with oligonucleotide, k_dim _equals 0.055 ± 0.016 and 0.30 ± 0.033 μM^-1^min^-1^, respectively (duplicate experiments).

To test whether formation of stem B directly affects SL1-mediated dimerization, we used the antisense oligonucleotides directed against the region upstream of SL1, including the 5' stem B (Figure 5A and Table 2). We previously showed that binding of the asΨ oligonucleotide increased the tight dimerization yield of 1–561 HIV-2 RNA by favoring SL1 presentation (Figure 3C and [26]). As described above, we used smaller RNAs so that the 3' end of the leader region (through CGI or LDI/BMH) would not influence tight dimerization. Although 1–444 HIV-2 RNA was shown to dimerize well in the absence of oligonucleotides ([20,22]), its rate more than doubled upon asΨ binding (Figures 5B and 5C). Interestingly, binding of the SIV asΨ oligonucleotide dramatically promoted the rate of SIV RNA dimer formation to a level similar to the one achieved using the asΨ oligonucleotide on HIV-2 RNA (18-fold increase; Figures 5B and 5C). Binding of the asΨ-homologous antisense oligonucleotide (as246) to 1–277 HIV-1 RNA also increased its tight dimerization rate five-fold, to a level similar to SIV and HIV-2 RNAs co-incubated with asΨ (Figures 5B and 5C).

Thus, binding of antisense oligonucleotides directed against the 5' side of stem B and nucleotides upstream increased the tight dimerization rate for all three lentiviral RNAs tested in this study. However, the rates without oligonucleotide differ and may be related to individual properties of the different lentiviral SL1s, including the apical loop, stem B, and adjacent sequences (Figure 4).

### Stability of stem B modulates the formation temperature of lentiviral RNA tight dimers

**Figure 6:**
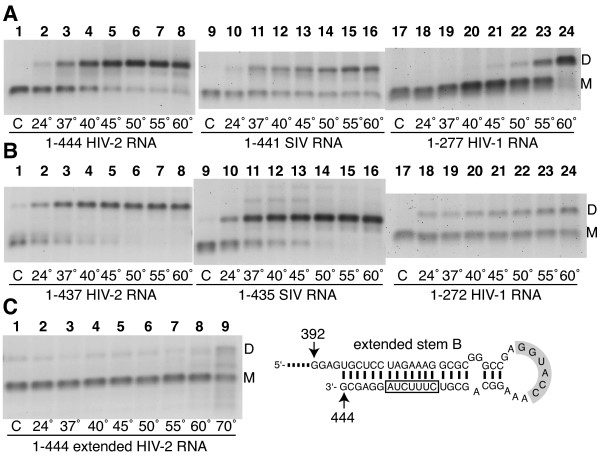
**Influence of incubation temperature and stem B truncation on lentiviral leader RNA tight dimerization**. The RNAs tested in panel **A **include the entire stem B (1–444 HIV-2, 1–441 SIV, and 1–277 HIV-1) and in panel **B **exclude the 3' side of stem B (1–437 HIV-2, 1–435 SIV, and 1–272 HIV-1) (see Figure 5, top panel, for structural details). **A and B**. All RNAs were individually incubated at the indicated temperatures (24°, 37°, 40°, 45°, 50°, 55°, and 60°C) for 30 min in their corresponding dimer buffer and subjected to electrophoresis on TBE agarose gel run at 26°C. Lane 1 represents monomeric RNA that was denatured at 90°C, then quenched on ice immediately prior to loading. **C**. A mutated 1–444 HIV-2 RNA with an extended SL1 was incubated at the indicated temperatures (24°, 37°, 40°, 45°, 50°, 55°, 60°, and 70°C) for 30 min in the dimer buffer H and subjected to electrophoresis on TBE agarose gel run at 26°C. The extension of SL1 is caused by the substitution of nts G^437^-U^438 ^by the sequence 5'-CUUUCUA-3' (right panel, boxed letters). This mutation doubles the stability of the SL1 region, from -18.2 to -36.4 kcal/mol (ΔGs of wild type and mutated 1–444 HIV-2 RNAs, respectively).

To examine the role of stem B stability on tight dimerization, we tested the influence of the incubation temperature on dimerization of RNA constructs ending after stem B (1–444 HIV-2, 1–441 SIV, and 1–277 HIV-1 RNAs) and in constructs lacking the 3' side of stem B (1–437 HIV-2, 1–435 SIV, and 1–272 HIV-1 RNAs). After denaturation and quench-cooling, the RNAs were incubated in dimer buffer for 30 minutes at temperatures ranging from 24° to 60°C (see Methods). The truncation of 3'-stem B induced a shift of the tight dimers temperature profile for all three RNAs (Figure 6). The temperature necessary to form 50% dimers after a 30-min incubation was between 37° and 40°C for 3'-stem-containing RNAs, but closer to room temperature (24°C) for RNAs missing 3'-stem B (Figure 6, compare panels A and B). The temperature effect suggests that disruption of stem B base pairing occurs during the formation of tight dimers.

Because disruption of stem B was shown to stimulate tight dimerization, we hypothesized that artificially stabilizing stem B would have the opposite effect. We thus substituted nts G^437^-U^438 ^in 1–444 HIV-2 RNA with the sequence CUUUCUA and called this mutant 1–444 extended SL1 HIV-2 RNA. This mutation extends the stem of the SL1 structure to include stem B through Watson-Crick base pairing (Figure 6C) and consequently doubles the stability of the SL1 region, from -18.2 to -36.4 kcal/mol (ΔGs of wild type and mutated RNAs, respectively; Table 3). The ΔG values correspond to the optimal secondary structure of 396–444 HIV-2 RNAs with or without the mutation using Mfold version 3.2 ([38,39]). The 1–444 HIV-2 RNA with the extended SL1 was incubated for 30 min at temperatures ranging from 24° to 70°C (Figure 6C). Its tight dimerization yields were very low at almost all temperatures; only the 70°C incubation showed an increase in dimer yield (Figure 6C). Thus, the increase in stability of the SL1 domain in the mutated 1–444 HIV-2 RNA correlated with a decrease in tight dimerization.

## Discussion

The leader RNA of lentiviruses contains signals involved in different stages of the replication cycle and most of these signals are conserved between primate lentiviruses. SL1 is involved in dimerization and encapsidation of the genomic RNA ([2,4,15]), and is located between the PBS domain and the major splice donor site in the 5' untranslated leader region of all spliced and unspliced lentiviral RNA species (Figure 1).

**Figure 7:**
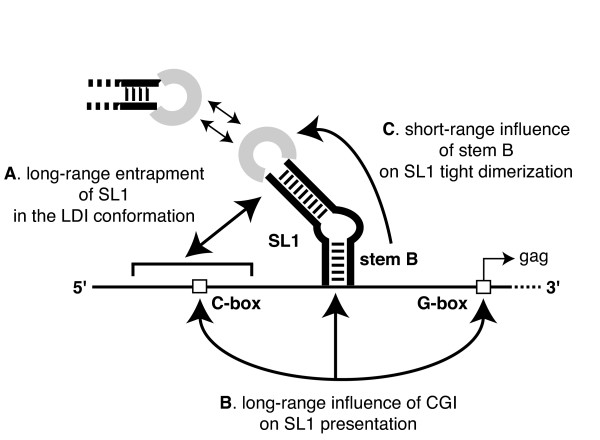
**Schematic of the different short- and long-range RNA interactions that regulate lentiviral SL1-mediated dimerization**. Three short- and long-range RNA structures can regulate SL1-mediated tight dimerization in HIV-2, SIV, and HIV-1. **A**. LDI (long-distance interaction) is an extensive, long-range base pairing interaction between the U5 and SL1 domains that causes a large rearrangement of the leader RNA structure [17]. SL1 is completely sequestered and thus unable to initiate dimerization. **B**. CGI (C-box – G-box interaction) is a long-range base pairing interaction between a sequence in U5 and the *gag *translation initiation region (called C-box and G-box, respectively). Formation of CGI impairs SL1-mediated dimerization in HIV-2 ([21,25]), and favors it in HIV-1 [17]. **C**. Stem B is the lower stem of SL1 and has important roles during viral replication in HIV-1 ([43,45]), HIV-2 ([6,7]), and SIV ([50,51]). Stem B-mediated SL1 entrapment may have twin roles in the regulation of lentiviral leader RNA dimerization since promoting SL1 presentation by increasing its folding stability (Table 3) may favor the formation of SL1 loop/loop (*i.e*. loose) dimers, while impairing the transition from loose to tight dimers.

The SL1 region of HIV-2 RNA has important roles in dimerization and encapsidation and we have documented its ability to be sequestered or presented both *in vitro *and during viral replication ([6,26,27]). Here we examined the dimerization properties of three primate lentiviral RNAs, HIV-2, SIV, and HIV-1, to search for common mechanistic themes in the regulation of SL1-mediated dimerization. The 5' untranslated leader RNA regions of HIV-2 ROD and SIV mac239 are similar in size and sequence [37], but our initial experiments showed that *in vitro *SIV RNA dimerization is slightly less efficient and, contrary to HIV-2 RNA, antisense oligonucleotides directed against CGI failed to enhance tight dimerization. Furthermore, different presentations of HIV-2 and SIV Ψ/SL1 region may partially explain the fact that they exhibit non-reciprocal cross packaging of genomic RNA in cell culture [40]. HIV-1 is phylogenetically more distant [37] and although its RNA dimerization properties have been more extensively studied, the present work aimed to directly compare dimerization regulation using standardized, homologous RNA constructs and experimental conditions. We demonstrated that SL1-mediated tight dimerization of three primate lentiviral RNAs can be regulated by a specific short-range (stem B) interaction and by a long-range interaction involving the 3' end of the leader RNA through either CGI-dependent (HIV-2) or CGI-independent (HIV-1 and SIV) mechanisms (Figure 7).

### Tight dimerization efficiency of primate lentiviral RNAs

We and others previously reported the low efficiency of HIV-2 leader RNA tight dimerization when its 3' end encompasses the *gag *initiation region ([21,25]). The impaired dimerization correlated with the formation of the CGI structure (Figure 1) ([21,25]). We proposed that the formation of HIV-2 CGI favors a structural intramolecular entrapment of SL1 during RNA folding and thus decreases its use as a tight dimerization signal *in vitro *(Figure 1D) ([25,26]).

Although extended kinetic analysis revealed an impaired dimerization phenotype of 1–550 SIV RNA, the lack of effect of antisense oligonucleotides directed against CGI underscored an unexpected difference between SIV and HIV-2 leader RNAs, despite their sequence homology. Indeed, CGI-independent intramolecular local folding events may compete with dimerization more effectively in SIV than in HIV-2 RNA. Similar to SIV, 1–373 HIV-1 RNA dimerization was not influenced by the binding of antisense oligonucleotides directed against CGI, although its yield without oligonucleotide was higher than HIV-2 or SIV. Notably, the dimerization rate for each RNA was markedly enhanced by truncation from the full-length leader to shorter versions that end after SL1 (compare Figures 2 and 5) suggesting a conserved modulatory role for downstream leader sequences.

### Stem B and lentiviral SL1-mediated dimerization

A unifying principle in the regulation of lentiviral RNA dimerization is the role of the lower stem of SL1, called stem B (Figures 5 and 7). We hypothesized that stem B exerts significant influence on lentiviral RNA dimerization for several reasons. First, stem B is highly conserved in all three lentiviral genomes sequences considered in this study ([22,41,42]). Second, recent reports have confirmed that short-distance interactions involving sequences located upstream of SL1 could regulate HIV-2 dimerization *in vitro *[27] and in cell culture [7]. Third, stem B was shown to have roles at several stages of HIV-1 replication, including genomic RNA dimerization ([43-45]).

In this study we showed that all three tested lentiviral species RNAs exhibited local regulation of dimerization through the stabilization of SL1 by stem B (Figure 5 and Table 3). We propose that stem B-mediated SL1 entrapment may have twin roles in the regulation of lentiviral leader RNA dimerization (Figure 7). First, it increases the probability of SL1 to fold as a discrete secondary structure, thus favoring SL1 loop/loop (loose) dimerization. It has been shown that HIV-1 stem B promotes SL1-mediated loose dimerization by favoring the correct presentation of the apical autocomplementary sequence ([43,46]). Second, promoting SL1 presentation by increasing its stability may have a thermodynamic cost for tight dimer formation. The thermodynamic cost might be compensated *in vivo *by the presence of the loop B structure in SL1. Indeed, several laboratories have shown that loop B (located between stems B and C, Figure 5A) favors temperature- or nucleocapsid-mediated dimerization by helping the transition from loose to tight dimers ([47-49]).

Several studies analyzed the roles of SL1 in genomic RNA dimerization and the nature of SL1–SL1 interaction *in vivo *but a comprehensive model has not yet been established, due to divergent results from several laboratories. SL1 was shown to promote HIV-1 (reviewed in [2]) and SIV ([50,51]) genomic RNA dimerization during viral encapsidation. Accordingly, Laughrea and colleagues proposed a model of HIV-1 genomic RNA dimerization wherein SL1 acts as a genomic RNA dimerization initiation signal that evolves to tight dimers [16]. Alternatively, Hu and colleagues showed that SL1 is involved in HIV-1 genomic RNA selection and recombination through intermolecular base pairing limited to SL1 loop-loop interaction [52]. It is possible that the apparent divergent results of both studies are due to different experimental conditions. Indeed, Laughrea and colleagues showed that SL1 is involved in the early stages of genomic RNA encapsidation, that is, in the first hours of viruses maturation after their release from the cell (five hours or less, [16]). They suggested that slow-acting dimerization site(s) mask the phenotype of SL1 mutants in "older" viruses (24 hours and older, [16]). Since Hu and colleagues harvested viruses after 24 hours of culture [52], the majority of their genomic RNAs may have been matured by slow-acting dimerization site(s), which may explain why most of their SL1 mutations were phenotypically silent. In HIV-2, Lever and colleagues showed that the HIV-2 palindromic sequence pal had more importance for genomic RNA dimerization during viral encapsidation than the palindromic sequence in the loop of SL1 [7].

Therefore, stem B of SL1 is likely a key regulating element for different conformations of lentiviral leader RNAs, akin to the concept of the riboswitch, proposed in HIV-1 by Berkhout and colleagues [3]. Disrupting stem B is detrimental to many stages of HIV-1 ([43,45]), HIV-2 ([6,7]), and SIV ([50,53]) replication. Remarkably, this extended SL1 structure means that there is a structural overlap between the Ψ encapsidation signal and the core of the SL1 dimerization element in both SIV and HIV-2. Recent reports of stem B effects on dimerization [7] and encapsidation [6] support a functional linkage between these two viral functions in HIV-2.

## Conclusion

This study demonstrates the ubiquity of structural entrapment as a mechanism of regulation of dimerization among primate lentiviruses and provides a framework for understanding dimerization data in each system through local and long-range RNA arrangements. The 3' leader region exerts an inhibitory effect on lentiviral RNA dimerization by altering the presentation of SL1. Stem B strongly influences SL1 presentation in all three lentiviral RNAs tested in this study. Because a similar set of folding rules can be used to explain the behaviors of three distinct primate lentiviral RNAs *in vitro*, it is likely that these discrete conformational changes represent a conserved regulatory mechanism *in vivo*.

In particular, it will be of interest to study the effect of CGI or stem B mutations on packaging and *gag *translation in mutant viruses in cell culture. Conformational changes like the ones described here may help determine the fate of viral RNA with respect to genomic and/or translational duties. Indeed, such viral riboswitches would be expected to be regulated by RNA chaperones like the viral nucleocapsid or Gag proteins and might be influenced by translational events, like ribosomal scanning.

## Abbreviations

HIV: Human immunodeficiency virus; SIV: Simian immunodeficiency virus; SL1: Stem-loop 1; CGI: C-box – G-box interaction; LDI: Long-distance interaction; BMH: Branched multiple-hairpin structure.

## Competing interests

The authors declare that they have no competing interests.

## Authors' contributions

JSL and JML designed the study. TTB, CLS, and JML carried out the experiments. JSL and JML drafted the manuscript. All authors read and approved the final manuscript.
